# Investigation of low 5-year relative survival for breast cancer in a London cancer network

**DOI:** 10.1038/sj.bjc.6605857

**Published:** 2010-08-24

**Authors:** E A Davies, K M Linklater, V H Coupland, C Renshaw, J Toy, R Park, J Petit, C Housden, H Møller

**Affiliations:** 1 King's College London, Thames Cancer Registry, 1st floor, Capital House, 42 Weston Street, London, UK; 2 North East London Cancer Network, First Floor, Outpatients Department, Royal London Hospital, Stepney Way, London E1 1BB, UK; 3 Roche Products Limited, 6 Falcon Way, Shire Park, Welwyn Garden City, AL7 1TW, UK

**Keywords:** breast cancer, stage, socioeconomic deprivation, survival, cancer network, cancer inequalities

## Abstract

**Background:**

Breast cancer 5-year relative survival is low in the North East London Cancer Network (NELCN).

**Methods:**

We compared breast cancer that was diagnosed during 2001–2005 with that in the rest of London.

**Results:**

North East London Cancer Network women more often lived in socioeconomic quintile 5 (42 *vs* 21%) and presented with advanced disease (11 *vs* 7%). Cox regression analysis showed the survival difference (hazard ratio: 1.27, 95% confidence interval (CI): 1.15–1.41) reduced to 1.00 (95% CI: 0.89–1.11) after adjustment for age, stage, socioeconomic deprivation, ethnicity and treatment. Major drivers were stage and deprivation. Excess mortality was in the first year.

**Conclusion:**

Late diagnosis occurs in NELCN.

Women living in socioeconomically deprived areas have a lower incidence of breast cancer (Shack *et al*, 2008), but a lower survival than those in affluent areas (Coleman *et al*, 2004). Variation in survival between English health authority areas is partly explained by their differing levels of deprivation (Mullee *et al*, 2004). Women from Black Caribbean and Black African ethnic groups have a lower incidence of breast cancer, but a lower survival than White women (Jack *et al*, 2009). These inequalities deserve investigation to identify factors amenable to intervention. For example, lower survival in one Swedish health district appeared to be because of a lower intensity of diagnostic tests influencing accurate staging and the treatment women received (Eaker *et al*, 2005). Investigations within English regions found that women living in deprived areas presented more often with advanced disease (Adams *et al*, 2004; Downing *et al*, 2007; Cuthbertson *et al*, 2009; Wishart *et al*, 2010). The lower survival of women from Black ethnic groups in South East England was largely explained by more advanced disease and socioeconomic deprivation (Jack *et al*, 2009).

This study investigates low breast cancer survival in North East London Cancer Network (NELCN). For the period 2001–2005, the 5-year relative survival was 75% (95% confidence interval (CI): 73.5–76.9) *vs* 79% (CI: 78.3–79.7) for London as a whole (Thames Cancer Registry, 2007b). North East London Cancer Network was also a low outlier for 1-year relative survival in England (National Cancer Intelligence Network, 2008). Our objectives were (1) to investigate whether differences in demographic, clinical or treatment characteristics between NELCN women and the rest of London explained this survival difference and (2) to identify clinical or public health approaches to improve survival.

## Materials and Methods

### Setting and hypotheses

NELCN covers a population of 1.5 million and is the most deprived of the London cancer networks. Informed by recent reports (Thames Cancer Registry, 2007a; Bowen *et al*, 2008; Greater London Authority, 2008), and clinical and public health experience, a multi-disciplinary team developed the following hypotheses to explain low survival: (1) low breast screening coverage led to a lower proportion of screen-detected cancers, (2) the diverse population included women from ethnic groups presenting with poorer prognosis cancers and (3) a higher proportion of patients lived in socioeconomically deprived areas.

### Data

The Thames Cancer Registry has received information on screen-detected breast disease from local screening programmes since 1998. We checked and extracted data on London women within the screening age group 50–64 years. We also extracted data on 3773 women of all ages diagnosed with invasive breast cancer resident in NELCN and on 17 059 women resident in the rest of London during 2001–2005. Self-assigned ethnicity information was obtained from Hospital Episode Statistics data, which includes ethnicity codes, as defined by the 1991 and 2001 censuses. Data for these years were linked to the Registry records and used to assign women to the categories White, Black, Asian, Chinese, Mixed and Other. Using postcode of residence at diagnosis, each woman was also assigned to a lower super output area and categorized to a quintile of deprivation using the Income Domain of the Index of Multiple Deprivation (Neighbourhood Renewal Unit, 2004).The Registry relies on data on disease stage recorded in individual medical records. This information is not always complete and for this study, all available Registry data on tumour size, lymph nodes or distant metastases were reviewed and used to assign a simplified TNM stage to each patient (Linklater and Møller, 2005). Data on whether the patient received surgery, chemotherapy, radiotherapy and hormonal treatment within the first 6 months after their diagnosis were also extracted.

### Analyses

As screen-detected cancers are more likely to be in earlier disease stage, the proportions of these cases in the screening age group for NELCN and the rest of London were compared. Five-year relative survivals for the period 2001–2005, comparing the survival of NELCN women with other London women for each of the screen-detected and non-screen-detected cancer diagnosed 1998–2005, were calculated. The age, socioeconomic deprivation, ethnicity, disease stage and treatment of women resident in NELCN and diagnosed during 2001–2005 were then compared with those in the rest of London. To assess influences on NELCN breast cancer-specific survival, we fitted Cox regression models, adjusting sequentially for age, stage, socioeconomic deprivation, ethnicity and treatment, comparing breast cancer-specific survival in NELCN women to all others in London. Finally, to determine the period in which excess mortality might be occurring, Kaplan–Meier survival curves for NELCN were constructed comparing it with the rest of London. We then redrew these survival curves for women who had already survived 1 year after diagnosis.

## Results

### Survival of women with screen-detected and non-screen-detected breast cancer in NELCN and the rest of London for the period 2001–2005

The proportion of NELCN women in the 50–64-year age group with screen-detected disease was slightly higher than for the rest of London (46 *vs* 43%). The 5-year relative survival for the period 2001–2005 of NELCN women with screen-detected disease was not significantly lower than those in the rest of London. However, the survival of NELCN women with non-screen detected cancer was lower (75.4% (CI: 72.0–78.8) *vs* 80.3% (CI: 78.9–81.5)). The proportions of screen-detected disease suggest that low screening uptake during 2001–2005 was not driving the overall NELCN survival difference. However, the lower survival for those with non-screen-detected disease suggests a group of NELCN women presenting with more advanced or aggressive disease.

### Characteristics of NELCN women compared with those in the rest of London

Table 1 shows the characteristics of all women resident in NELCN and diagnosed with breast cancer between 2001 and 2005 compared with those in the rest of London. Age and ethnicity did not differ substantially, but NELCN women were more likely to be living in the most deprived areas (quintile 5) (42 *vs* 21%). North East London Cancer Network women were also more likely to be diagnosed with stage 4 disease (metastases; 11 *vs* 7%) and were less likely to receive radiotherapy (25 *vs* 31%).

### Contribution of case mix factors to the lower survival in NELCN

Table 2 shows the Cox proportional regression analysis. The hazard ratio (relative risk of mortality) for patients in NELCN was 1.27. After adjustment for age, this risk reduced slightly to 1.23. The excess risk was then halved and reduced to 1.12 by adjustment for stage, and substantially reduced again to 1.04 by adjustment for socioeconomic deprivation. Further adjustment for ethnicity made no difference to the remaining risk, but the subsequent addition of treatment reduced the hazard ratio to 1.00. These results indicate that the main drivers of the excess mortality in NELCN women were more advanced stage of disease at diagnosis and higher levels of deprivation. We interpreted the small effect of treatment as a further indicator of advanced disease stage, rather than of under-treatment.

### Timing of excess risk of mortality

**Figure 1:**
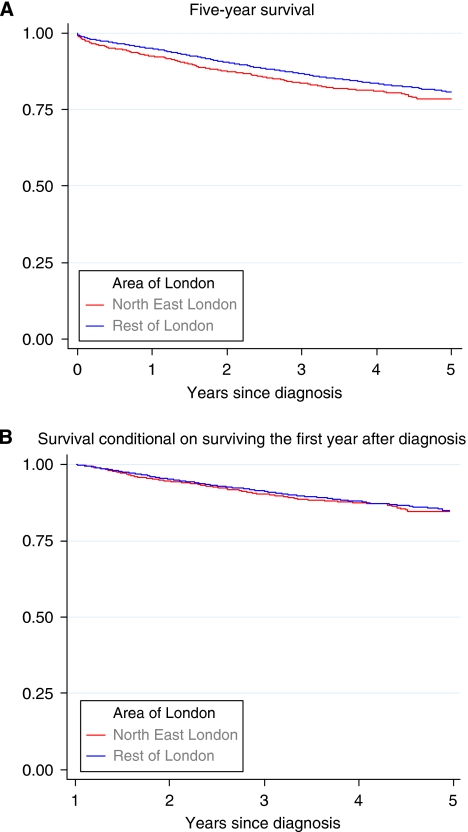
Kaplan–Meier survival estimates for NELCN women with breast cancer compared with women in the rest of London, 2001–2005. (**A**) 5-year survival. (**B**) Survival conditional on surviving the first year after diagnosis.

The Kaplan–Meier survival curves for NELCN women with breast cancer compared with those in the rest of London are shown in Figure 1A. The curves show an early divergence and then remain parallel. The survival curves for patients still alive at 1 year after the diagnosis are very similar (Figure 1B). This demonstrates that the excess deaths in NELCN occur in the first year after diagnosis, suggesting late diagnosis.

## Discussion

### Summary of main findings

NELCN women with breast cancer more commonly presented with advanced disease and lived in areas of higher socioeconomic deprivation, and these factors appear to be the main causes of their higher mortality. The excess risk occurred in the first year after diagnosis, suggesting, given the usual growth behaviour of breast cancer, that women dying in this period had advanced disease at diagnosis for which treatments have limited effectiveness in extending the survival. This strongly suggests the need to develop socially targeted strategies to promote earlier diagnosis. In this regard, it is noteworthy that when only screen-detected breast cancer was considered, there was no significant 5-year relative survival difference between NELCN women and those in the rest of London.

### Comparison with other findings

Socioeconomic deprivation is well established to be associated with lower breast cancer survival (Adams *et al*, 2004; Mullee *et al*, 2004; Downing *et al*, 2007; Cuthbertson *et al*, 2009), but can only be a relatively crude indicator of other factors, including lower education and awareness of breast cancer (Robb *et al*, 2009), lower screening uptake (Moser *et al*, 2009), differing behaviour in response to symptoms (Ramirez *et al*, 1999), competing comorbidities and differing access to primary care and referral. The recently published NHS ‘*All Breast Cancer Report*’ showed a significant 3.6% difference in 1-year relative survival between the most deprived and most affluent symptomatic women diagnosed in 2001–2002, but showed no significant difference in women with screen-detected cancers. There was a 12.2% difference in 5-year survival between the most deprived and most affluent quintiles for symptomatic cancers and a much smaller one (6.6%) for screen-detected cancers (National Health Service Cancer Screening Programmes, 2009). More data on screening uptake and screening histories would have been helpful. The similar proportion of screen-detected cancer in NELCN and the rest of London suggests similar screening uptake, although some differences might have been expected given the higher deprivation in NELCN and the strong association of deprivation with lower uptake in London (Renshaw *et al*, 2010).

Presentation with more advanced disease has a major role in explaining the lower survival of English women with breast cancer (Richards, 2009). Excess mortality in English women compared with Swedish and Norwegian women occurs mostly in the first 12 months after diagnosis, particularly in older patients. After this time, the differences diminish, suggesting that later diagnosis, rather than less effective treatment, in England might be responsible (Møller *et al*, 2010). A similar mechanism of later diagnosis is suggested for the excess 1-year mortality in NELCN women of all ages. It is possible that lower survival in English women could, in part, be driven by later diagnosis in areas of higher socioeconomic deprivation as identified in London.

### Implications

This study reinforces the importance of not making assumptions about cause without investigation. It also shows how comparative cancer survival figures can generate local initiatives. North East London Cancer Network has set a target to improve breast cancer survival to London levels by 2012 and a new Public Health Advisory Board steers a programme to promote earlier diagnosis. Initiatives include social marketing interventions to increase screening uptake, encouraging all women with symptoms to present early, audit of deaths within a year of diagnosis and an education campaign to improve public awareness of early cancer symptoms. Surveys will assess population breast cancer awareness to evaluate the effect of these interventions. There is more research on interventions to promote screening uptake (Bonfill Cosp *et al*, 2001) than on earlier diagnosis of symptomatic disease. Catalano *et al* (2003) found that repeated breast cancer awareness campaigns could promote earlier diagnosis. This investigation emphasises the need to understand why socioeconomic deprivation is associated with poor survival and to develop creative methods to engage with local communities (Eilbert *et al*, 2009; Lyon *et al*, 2009).

### Limitations

Under ascertainment of better prognosis breast cancers could lead to incorrect low relative survival figures. Three findings argue against this: (1) breast cancer incidence in each quintile of deprivation within NELCN was very similar to those in the rest of South East England, (2) the breast screening programme did not supply a large number of extra cases and (3) the number of cases recorded by the Registry was similar to that within NELCN clinical information systems. The staging data available for this study were incomplete with 26–30% of cases unknown. Audit of network clinical systems and medical records found that data were also incomplete. Work on improved collection of staging and comorbidity data at multi-disciplinary team meetings, and its transfer to the Registry is underway.

## Figures and Tables

**Table 1:** Demographic and clinical characteristics of NELCN women with breast cancer compared with the rest of London

**Breast cases**	**NELCN**		**Rest of London**	
**Females**	**No. 3773**	**%**	**No. 17 059**	**%**
Age group				
00–49	850	22.5	4033	23.6
50–64	1252	33.2	6254	36.7
65–74	718	19.0	3029	17.8
75–84	664	17.6	2519	14.8
85+	289	7.7	1224	7.2
				
*SES*				
1 (Affluent)	216	5.7	2764	16.2
2	412	10.9	2968	17.4
3	606	16.1	3501	20.5
4	947	25.1	4276	25.1
5 (Deprived)	1592	42.2	3550	20.8
				
*Stage*				
Stage 1	1038	27.5	4106	24.1
Stage 2–3	1274	33.8	6383	37.4
Stage 4	430	11.4	1115	6.5
Not known	962	25.5	5041	29.6
				
*Treatment*				
Any surgery	3173	84.1	14 273	83.7
Any chemotherapy	906	24.0	4745	27.8
Any hormone	1118	29.6	5832	34.2
Any radiotherapy	958	25.4	5310	31.1
No treatment	332	8.8	1198	7.0
DCO	69	1.8	414	2.4
				
*Ethnic group*				
White	2441	64.7	10 662	62.5
Asian	247	6.5	842	4.9
Black	245	6.5	1022	6.0
Mixed	15	0.4	63	0.4
Chinese	16	0.4	82	0.5
Other	69	1.8	561	3.3
Not known	740	19.6	3827	22.4

Abbreviations: DCO=Death Certificate Only; NELCN=North East London Cancer Network; SES=Socioeconomic Status.

**Table 2:** Hazard ratios for breast cancer specific mortality for women diagnosed 2001–2005 in NELCN compared with the rest of London

	**Univariate**	**Adjusted for age**	**Adjusted for age, stage**	**Adjusted for age, stage, deprivation**	**Adjusted for age, stage, deprivation, ethnicity**	**Adjusted for age, stage, deprivation, ethnicity, treatment**
**Covariate**	**Hazard ratio (95% confidence interval)**	**Hazard ratio (95% confidence interval)**	**Hazard ratio (95% confidence interval)**	**Hazard ratio (95% confidence interval)**	**Hazard ratio (95% confidence interval)**	**Hazard ratio (95% confidence interval)**
*NELCN*						
Rest of London^a^	1.00	1.00	1.00	1.00	1.00	1.00
NELCN	1.27 (1.15–1.41)	1.23 (1.11–1.37)	1.12 (1.01–1.24)	1.04 (0.94–1.16)	1.05 (0.94–1.17)	1.00 (0.89–1.11)
						
*Age Group*						
<50^a^		1.00	1.00	1.00	1.00	1.00
50–64		0.88 (0.77–1.01)	1.13 (0.99–1.30)	1.16 (1.02–1.33)	1.19 (1.04–1.36)	1.27 (1.11–1.46)
65–74		1.73 (1.51–1.99)	1.82 (1.59–2.10)	1.87 (1.63–2.15)	1.92 (1.67–2.22)	2.47 (2.13–2.86)
75–84		2.90 (2.53–3.31)	2.83 (2.47–3.24)	2.90 (2.53–3.32)	3.02 (2.62–3.47)	4.23 (3.63–4.93)
85+		7.72 (6.66–8.95)	6.54 (5.64–7.59)	6.66 (5.74–7.74)	6.95 (5.95–8.11)	9.19 (7.76–10.88)
						
*Stage*						
Stage 1^a^			1.00	1.00	1.00	1.00
Stage 2–3			3.92 (3.18–4.82)	3.89 (3.16–4.78)	3.87 (3.14–4.76)	3.39 (2.75–4.17)
Stage 4			35.08 (28.53–43.13)	34.71 (28.23–42.68)	34.46 (28.02–42.37)	27.27 (22.11–33.63)
Not known			6.02 (4.89–7.40)	6.09 (4.95–7.49)	6.07 (4.93–7.46)	4.60 (3.73–5.67)
						
*Quintile of deprivation*						
1 (Affluent)^a^				1.00	1.00	1.00
2				1.13 (0.96–1.34)	1.13 (0.95–1.34)	1.13 (0.95–1.34)
3				1.36 (1.16–1.59)	1.35 (1.15–1.58)	1.36 (1.16–1.60)
4				1.40 (1.20–1.63)	1.38 (1.18–1.60)	1.41 (1.21–1.65)
5 (Deprived)				1.53 (1.31–1.78)	1.49 (1.28–1.74)	1.45 (1.24–1.70)
						
*Ethnic Group*						
White^a^					1.00	1.00
Asian					1.11 (0.89–1.37)	1.10 (0.89–1.36)
Black					1.22 (1.02–1.47)	1.16 (0.97–1.39)
Mixed					2.13 (1.26–3.63)	2.16 (1.27–3.67)
Chinese					0.35 (0.11–1.08)	0.38 (0.12–1.20)
Other					1.20 (0.93–1.56)	1.05 (0.81–1.37)
Not known					1.05 (0.95–1.17)	1.04 (0.93–1.15)
						
*Had surgery*						
N^a^						1.00
Y						0.52 (0.46–0.57)
						
*Had chemotherapy*						
N^a^						1.00
Y						1.54 (1.38–1.72)
						
*Had radiotherapy*						
N^a^						1.00
Y						0.69 (0.62–0.76)
						
*Had hormone therapy*						
N^a^						1.00
Y						0.74 (0.67–0.82)

Abbreviations: N=no; NELCN=North East London Cancer Network; Y=yes.

a Baseline category.
